# Association Between Nonselective Beta-Blocker Use and Hepatocellular Carcinoma in Patients With Chronic Hepatitis B Without Cirrhosis and Decompensation

**DOI:** 10.3389/fphar.2021.805318

**Published:** 2022-01-07

**Authors:** He-Yun Cheng, Hsiu C. Lin, Hsiu L. Lin, Yow S. Uang, Joseph J. Keller, Li H. Wang

**Affiliations:** ^1^ School of Pharmacy, College of Pharmacy, Taipei Medical University, Taipei, Taiwan; ^2^ Department of Pediatrics, School of Medicine, College of Medicine, Taipei Medical University, Taipei, Taiwan; ^3^ Department of Clinical Pathology, Taipei Medical University Hospital, Taipei, Taiwan; ^4^ Department of Neurology, General Cathay Hospital, New Taipei City, Taiwan; ^5^ College of Medicine, Ohio State University, Columbus, OH, United States; ^6^ Department of Pharmacy, Taipei Medical University Hospital, Taipei, Taiwan

**Keywords:** nonselective beta-blockers, hepatocellular carcinoma, chronic hepatitis B, hepatitis B infection, propranolol, carvedilol, liver cancer

## Abstract

**Background:** Nonselective beta-blockers (NSBBs) can reduce the incidence or mortality of certain types of cancers, and NSBBs exert a protective effect on hepatocellular carcinoma (HCC) in patients with cirrhosis. However, the potential preventive effect of NSBBs has not yet been investigated in patients with chronic hepatitis B (CHB) who have a high HCC risk regardless of the presence of underlying cirrhosis.

**Aim:** This study evaluated the association between NSBB use and HCC incidence in patients with CHB without cirrhosis and decompensation.

**Methods:** From the 2000 Longitudinal Generation Tracking Database, we enrolled patients who were newly diagnosed as having CHB from January 2001 to December 2011 and then followed them up for at least 5 years. To estimate the causal effect of NSBBs on the time-to-event outcomes of HCC, a marginal Cox proportional hazards model was used to calculate hazard ratios (HRs) and 95% confidence intervals (CIs).

**Results:** After adjustment, no significant benefit of HCC risk reduction was observed between the NSBB users and nonusers (adjusted HR, 0.82; 95% CI, 0.52–1.31). The cumulative defined daily dose (cDDD) analysis revealed no significant dose correlation among the three groups [adjusted HR (95% CI): 1.08, (0.56–2.05), 0.54 (0.17–1.77), and 0.76 (0.40–1.42) in the <90 cDDD, 90 to <180 cDDD, and ≥180 cDDD groups, respectively]. Duration-dependent associations were not observed. Multivariable stratified analysis results demonstrated that HCC risk markedly decreased in the patients aged >55 years (adjusted HR, 0.49; 95% CI, 0.25–0.96; *p* = 0.04).

**Conclusion:** NSBB did not significantly prevent HCC in the patients with CHB infection without cirrhosis and decompensation. This study provided one of valuable results that it is not clinically required to use NSBBs as recommended chemoprevention for HCC in high-risk patients who have CHB.

## 1 Introduction

Hepatocellular carcinoma (HCC) is the sixth most common cancer worldwide, accounting for >80% of primary liver malignancies (Yang et al., 2019). The incidence and mortality of HCC remain high, and HCC is estimated to cause >1 million deaths in 2030 (Villanueva, 2019). Hepatitis B virus (HBV) infection is a crucial risk factor for HCC (Thiele et al., 2014). Although HBV vaccination can reduce the risk of chronic hepatitis B (CHB), numerous unvaccinated individuals and HBV carriers still have a risk of HCC (Villanueva, 2019). In addition, patients who exhibit HBV surface antigen (HBsAg) clearance after receiving nucleotide analogue (NA) therapy still have a risk of HCC (Kim et al., 2015; Papatheodoridis et al., 2015). HCC is mostly diagnosed at a late stage, thus resulting in limited treatment options and poor prognosis. Hence, effective strategies must be developed to prevent HCC in patients with CHB.

Trends in drug repurposing have led to an increase in the number of studies exploring the efficacy of chemopreventive drugs in various cancers. Clinical and human studies have reported that nonselective beta-blockers (NSBBs) reduced the incidence or mortality of the cancers of the upper gastrointestinal tract and other types of solid cancers (Chang et al., 2015; Lin et al., 2015; Pantziarka et al., 2016) The anticancer effects of NSBBs were demonstrated by the finding that a long treatment with propranolol, an NSBB, reduced mortality in patients with unresectable and metastatic HCC in a duration-dependent manner (Chang et al., 2019). In human liver cancer cell lines, propranolol inhibited cancer cell proliferation by inducing apoptosis and S-phase arrest (Wang et al., 2018). This effect of propranolol may be attributable to the inhibition of beta-2 adrenergic receptors, reduction of bacterial translocation, and inhibition of fibrosis or angiogenesis through the downregulation of the vascular endothelial growth factor (Hamdy and El-Demerdash, 2012; Kassahun et al., 2012; Zhang et al., 2012; Moriya and Minamino, 2017). For patients with cirrhosis and oesophageal varices, NSBBs are recommended for the primary and secondary prevention of variceal bleeding (Tripathi, 2012; Garcia-Tsao et al., 2017). Some clinical studies have reported that NSBBs exert pleiotropic effects such as reducing the risk of HCC in patients with liver cirrhosis (Nkontchou et al., 2012; Thiele et al., 2015; Herrera et al., 2016; Wijarnpreecha et al., 2021).

Clinical studies have reported controversial findings regarding the role of NSBBs in HCC prevention, and most studies have recruited only patients with cirrhosis as the study population (Kim et al., 2012; Hagberg et al., 2016; Yeh et al., 2019; Wijarnpreecha et al., 2021). No study has examined the association between NSBBs and the incidence of HCC in patients with CHB who may develop HCC without cirrhosis and have a lower risk of HCC than do patients with existing cirrhosis. In addition, most studies have recruited a Western population, whose aetiology of HCC and responses to NSBBs differ from those of the East Asian population (Zhou et al., 1989; Hu et al., 2007). Therefore, this study investigated the association between NSBB use and the incidence of HCC in patients with CHB in the absence of liver cirrhosis and decompensation.

## 2 Methods

### 2.1 Data Source

Taiwan’s National Health Insurance (NHI) program covers 99.99% of Taiwan’s population. The claims data of NHI beneficiaries are collected and added to databases by the Health and Welfare Data Science Center (HWDC) of the Ministry of Health and Welfare. We used data from the 2000 Longitudinal Generation Tracking Database (LGTD 2000), which contains the information of 2 million beneficiaries randomly sampled from the NHI database. This subset contains comprehensive information regarding patients’ demographic variables such as age, sex, outpatient visits, and hospitalisations; disease diagnoses; procedure codes; prescription details; and healthcare item costs (Lin et al., 2018). This study also used the Registry for Catastrophic Illness Patients Database. To deduct certain NHI payments and copayments, patients with malignancies are required to apply for the catastrophic illness certification. All patient applications include complete histopathological or imaging confirmation from physicians and are formally reviewed by experts (Hsieh et al., 2019). Therefore, cancer diagnoses in the Registry for Catastrophic Illness Patients Database are highly accurate and can be used for validation.

Datasets released by the HWDC are anonymised and encrypted to protect patients’ privacy; thus, researchers cannot identify individuals. This study was approved by the Joint Institutional Review Board and Ethics Committee (JIRB) of Taipei Medical University, Taipei, Taiwan (TMU-JIRB No. 201909025). The requirement for patients’ informed consent was waived.

### 2.2 Study Design and Population

In this population-based retrospective cohort study, we enrolled patients who were newly diagnosed as having CHB [*International Classification of Diseases*, *Ninth Revision, Clinical Modification* (*ICD-9-CM*) codes: 070.2, 070.3, and V02.61] recorded at least three times in outpatient clinics or once during hospitalization from January 1, 2001, to December 31, 2011. The index date was the first prescription date of an NSBB in the NSBB use cohort and the matched date in the nonuse cohort (Supplementary Figure S1). The washout period for examining baseline characteristics was 1 year before the index date. To ensure the inclusion of the exposure time window and reduce immortal time bias, we included a follow-up period from the 180th day of the index date until HCC diagnosis, liver transplantation, death, or the end of the study period, whichever occurred first.

**FIGURE 1:**
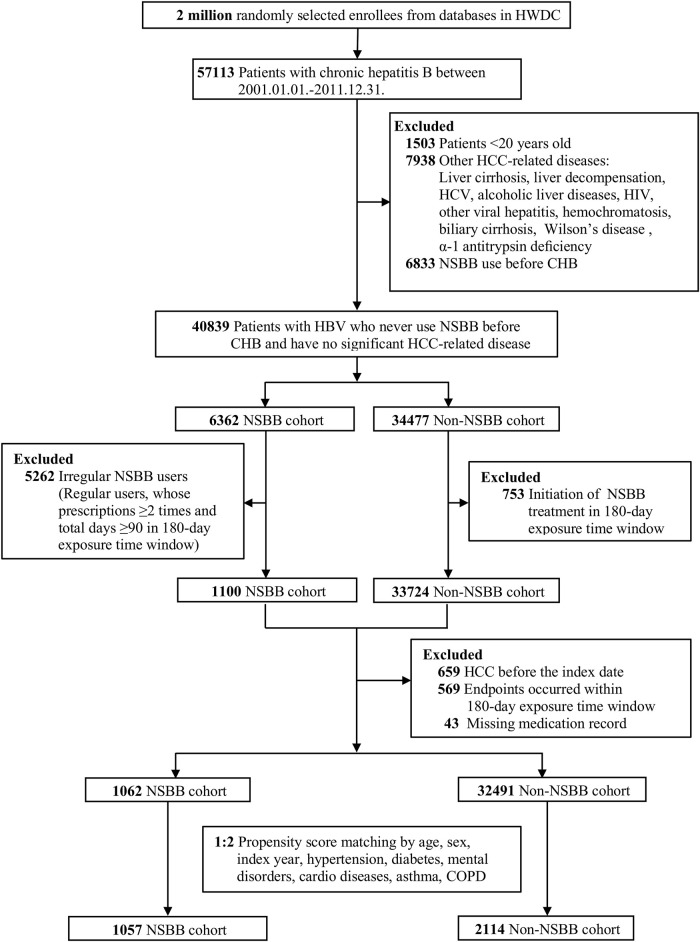
Flow chart of study population selection. HWDC, Health and Welfare Data Science Center; HCC, hepatocellular carcinoma; HCV, hepatitis C virus; HIV, human immunodeficiency virus; NSBB, nonselective beta blocker; CHB, chronic hepatitis B; COPD, chronic obstructive pulmonary disease.

Figure 1 depicts the selection process of the study population. Patients aged <20 years were excluded. We also excluded individuals with a history of specific HCC-related diseases such as liver cirrhosis, liver decompensation, hepatitis C virus infection, other viral hepatitis, human immunodeficiency virus infection, alcoholic liver disease, haemochromatosis, biliary cirrhosis, Wilson’s disease, and alpha-1 antitrypsin deficiency. To apply a new user design, patients who were prescribed with NSBBs more than once before the CHB diagnosis were excluded. In addition, we excluded those who irregularly used NSBBs and nonusers who initiated NSBB treatment during the exposure time window. We excluded patients who had HCC before the index date, patients who met an endpoint (HCC, liver transplantation, or death) within the 180-days exposure period, and patients with missing medication records. A 1:2 propensity score matching was employed to ensure the comparability of the two study groups and reduce selection and confounding biases (Sturmer et al., 2014). Each NSBB user was randomly propensity score–matched at a ratio of 1:2 with NSBB nonusers on the basis of several sociodemographic variables related to the likelihood of NSBB treatment, namely age, sex, calendar year of the index date, hypertension, diabetes, mental disorders, cardiac diseases, asthma, and chronic obstructive pulmonary disease.

### 2.3 Definition of Exposure

Patients who received NSBBs were identified from the database by using Anatomical Therapeutic Chemical (ATC) codes for nonselective beta-blocking agents (C07AA) and alpha- and beta-blocking agents (C07AG). Regular NSBB users were defined as those who received at least two prescriptions of NSBBs in the outpatient clinic and consumed the medication for >90 days during the 180-days exposure time window since the index date. Irregular users were defined as those who did not meet both the above-mentioned criteria. Patients who never received NSBBs during the enrolment period were included in the nonuser cohort. Alpha- and beta-blocking agents exert nitric oxide–generating and antioxidative effects that may be more beneficial to treating cancer than beta-blocking agents alone ( Fonseca, 2010; Hickok and Thomas, 2010; Thyagarajan and Sahu, 2018). On the basis of pharmacological mechanisms, NSBBs were classified into only beta-blocking agents (alprenolol, bupranolol, carteolol, nadolol, oxprenolol, pindolol, propranolol, sotalol, and timolol) and alpha- and beta-blocking agents (carvedilol and labetalol). In terms of chemical properties, carvedilol has a longer half-life and lower lipophilicity than does propranolol. Because of variations in the properties of individual NSBBs, we performed another subgroup analysis based on the use of specific NSBBs (propranolol and carvedilol). Patients who received monotherapy with a certain drug throughout the enrolment period were included in the subgroups.

### 2.4 Covariates and Confounding Factors

Covariates related to HCC were comorbidities, lifestyle exposure, metabolic factors, and medication use. We identified the following comorbidities at the index date: hypertension, hyperlipidaemia, diabetes, and nonalcoholic liver diseases. In addition, some potential chemopreventive agents with robust evidence, namely antiviral therapy (NAs and interferons), aspirin, statin, and metformin, were considered covariates. Lifestyle factors, namely tobacco use, alcohol use, and obesity, were assessed and defined on the basis of physicians’ diagnoses in the databases. Diagnoses of these coexisting diseases for adjustment were considered if recorded more than two times during outpatient visits and once during hospitalization. Except for antiviral therapy, medication use was identified on the basis of a 28-days prescription within 1 year before the index date. The users of antiviral agents were defined as those who received antiviral agents for >1 week. In accordance with the reimbursement criteria of NAs and interferons in Taiwan, patients must reach a certain CHB severity level to be eligible for the reimbursement; for example, they must have an elevated alanine aminotransferase level of at least two times the two-fold upper limit of the normal level in combination with ≥2,000 copies/ml of HBV DNA. The prescription of these two drugs may reflect the disease severity of patients with CHB. Hence, the prescriptions of NAs and interferon were used as a surrogate variable for the adjustment of disease severity. To identify differences in covariates between the NSBB users and nonusers, the standardised mean difference (SMD) was used to balance diagnostics after propensity score matching ( Austin, 2009 ). An absolute value of SMD greater than 0.10 (small effect size) indicated meaningful imbalance. Imbalanced variables were adjusted in the Cox regression model ( Nguyen et al., 2017 ).

### 2.5 Outcome Measurement

#### 2.5.1 Primary Outcome

The primary outcome was incident HCC. The diagnosis of HCC obtained from outpatient and inpatient records was identified on the basis of *ICD-9-CM* code 155 and *International Classification of Diseases, Tenth Revision, Clinical Modification* (*ICD-10-CM*) code C22. Patients with a diagnosis of HCC were also identified using the Registry for Catastrophic Illness Patient Database. Censored events in this study included liver transplantation and death. Liver transplantation was confirmed on the basis of the diagnosis of liver transplant status (*ICD-9-CM* code V42.7 and *ICD-10-CM* code Z94.4) or liver transplant surgery (procedure codes 505, 75020A, or 75020B). The Cause of Death Database, which contains information regarding the cause and date of death, was used to confirm deaths in the study population.

#### 2.5.2 Secondary Outcome

Secondary outcomes included dose- and duration-dependent associations between NSBB use and HCC incidence, effect of pharmacological classes and individual NSBBs, and subgroups by baseline characteristics. We used the ATC and defined daily dose (DDD) to explore drug use in the population. The DDD of each NSBB in this study was based on the treatment of mild-to-moderate hypertension. In the subgroup analysis, the NSBB use group was categorised by their cumulative DDDs (cDDDs) into <90, 90–180, and ≥180 cDDD groups. The duration of medication use in the NSBB users was determined through the summation of all the intervals of NSBB use during the follow-up period. On the basis of pharmacological mechanism, NSBBs were classified as beta-blocking agents or alpha- and beta-blocking agents in the subgroup analysis. Because of variations in the properties of individual NSBBs, we examined propranolol and carvedilol use in one subgroup analysis.

### 2.6 Statistical Analysis

Baseline continuous variables are reported as the mean ± standard deviation. Categorical variables are reported as the percentage. The survival curves of cumulative HCC incidence were plotted using the Kaplan–Meier method. Differences between the two curves were examined using the log-rank test. To determine the causal effect of NSBBs on the time-to-event outcomes of HCC incidence, a marginal Cox proportional hazard model was used to calculate hazard ratios (HRs) and 95% confidence intervals (CIs) (Chen et al., 2010; Austin, 2014). In the marginal Cox proportional hazard model, a robust sandwich covariance matrix estimator was used to account for clustering and produce unbiased HRs, which have a precise standard error. Univariable analysis was performed to examine potential risk factors for HCC in this population. Multivariable analysis was performed to determine the effect of NSBBs on HCC. Different variables were adjusted in the regression models: 1) age and sex; 2) age, sex, and comorbidities (namely hypertension, hyperlipidaemia, diabetes, and nonalcoholic liver diseases); 3) age, sex, comorbidities, and medication use (namely antiviral therapy, statin, metformin, and aspirin); 4) age, sex, comorbidities, medication use, and lifestyle (namely alcohol use, tobacco use, and obesity); and 5) imbalanced baseline variables that were tested on the basis of the SMD (Nguyen et al., 2017). To examine the subgroups of patients classified by baseline characteristics, we performed multivariable stratified analysis. To investigate the effect of pharmacological classes and individual NSBBs, the classified users were compared with their paired controls, and HRs were adjusted for unbalanced variables. To test the proportional hazards assumption, we determined the significance of the time-dependent explanatory variable and observed no violation of the proportional hazards assumption (Kleinbaum and Klein, 2012).

In the sensitivity analysis, we divided the study into three parts to evaluate the rigidity of the results. First, to reduce immortal time bias (from CHB diagnosis to NSBB prescription), we assigned the NSBB nonusers index dates by matching them on the basis of year of CHB diagnosis. Second, we excluded patients with prior selective beta-blocker use. Lastly, because the effect of NSBBs on HCC prevention may not persist for >10 years, we followed up with every participant for 10 years from their index dates. All data analyses were performed using SAS (version 9.4, SAS Institute Inc., Cary, NC, United States). Statistical significance was defined as a two-tailed *p* value of <0.05.

## 3 Results

### 3.1 Demographic Characteristics of the Study Population

Figure 1 presents the study population. We identified 1,062 patients with NSBB use and 32,491 patients without NSBB use between January 1, 2001, and December 31, 2011. After the propensity score matching of the 3,171 patients recruited in this study, we determined that the mean age of the patients was 51.35 (±13.36) years, and 1,878 (57.41%) of them were men. In total, 1,057 patients who used NSBBs were included in the NSBB use group, and 2,114 patients who did not use NSBBs were included in the non–NSBB use group. Table 1 lists the demographic characteristics of the participants. Overall, 83 (2.62%) patients received a diagnosis of HCC [25 (2.37%) and 58 (2.74%) in the NSBB user and nonuser cohorts, respectively]. The follow-up durations of the two groups were similar during a median follow-up period of 8.18 years (median, 8.18 years; range, 6.30–10.60 years). Most of the patients were followed until the end of the study period (2,826 cases, 89.12%), with few censored owing to death (262 cases, 8.26%). No patient was censored because of liver transplantation.

**TABLE 1:** Demographic characteristics of participants.

	Full cohort			1:2 propensity score–matched cohort^a^		
Variables	NSBB use (*n* = 1,062)	No NSBB use (*n* = 32,491)	SMD^b^	NSBB use (*n* = 1,057)	No NSBB use (*n* = 2,114)	SMD^b^
Age, years, mean ± SD	51.42 ± 13.37	41.86 ± 13.47	0.71	51.38 ± 13.36	51.33 ± 13.37	0.00
Sex/male, *n* (%)	624 (58.76)	20,465 (62.99)	−0.09	620 (58.66)	1,258 (59.51)	−0.02
Comorbidities, *n* (%)						
Hypertension	512 (48.21)	3,598 (11.07)	0.89	507 (47.97)	1,080 (51.09)	−0.06
Hyperlipidaemia	205 (19.30)	2,360 (7.26)	0.36	202 (19.11)	379 (17.93)	0.03
Diabetes	188 (17.70)	2,264 (6.97)	0.33	188 (17.79)	357 (16.89)	0.02
Mental disorders	273 (25.71)	2044 (6.29)	0.55	268 (25.35)	545 (25.78)	−0.01
Cardiac diseases	277 (26.08)	1,332 (4.10)	0.65	272 (25.73)	460 (21.76)	0.09
Asthma	26 (2.45)	566 (1.74)	0.05	25 (2.37)	66 (3.12)	0.05
COPD	53 (4.99)	687 (2.11)	0.16	51 (4.82)	84 (3.97)	0.04
Tobacco use	5 (0.47)	85 (0.26)	0.03	5 (0.47)	21 (0.99)	−0.06
Alcohol use	5 (0.47)	85 (0.26)	0.03	5 (0.47)	14 (0.66)	−0.03
Obesity	7 (0.66)	91 (0.28)	0.06	7 (0.66)	9 (0.43)	0.03
Nonalcoholic liver diseases	22 (2.07)	448 (1.38)	0.05	22 (2.08)	42 (1.99)	0.01
Medication use, *n* (%)						
Antiviral therapy^c^	22 (2.07)	238 (0.73)	0.11	22 (2.08)	26 (1.23)	0.07
Statin	118 (11.11)	1,011 (3.11)	0.32	118 (11.16)	199 (9.41)	0.06
Metformin	114 (10.73)	1,308 (4.03)	0.26	114 (10.79)	217 (10.26)	0.02
Aspirin	202 (19.02)	999 (3.07)	0.53	200 (18.92)	285 (13.48)	0.15
Possible reasons for NSBB use, *n* (%)						
Hypertension	449 (42.28)	NA	NA	447 (42.29)	NA	NA
Cardiac diseases	339 (31.92)	NA	NA	337 (31.88)	NA	NA
Mental disorders	232 (21.85)	NA	NA	229 (21.67)	NA	NA
Migraine	17 (1.60)	NA	NA	17 (1.61)	NA	NA

NA, not available; COPD, chronic obstructive pulmonary disease; NSBB, nonselective beta-blocker; SD, standard deviation.

a Propensity score matched by age, sex, calendar year of the index date, hypertension, diabetes, mental disorders, cardiac diseases, asthma, and COPD.

b Standardised mean difference (SMD) = difference in means or proportions divided by the standard error; imbalance defined as an absolute value of >0.10 (small effect size).

c Antiviral therapy includes nucleos(t)ide analogues and interferon therapies.

### 3.2 Primary Outcome

**FIGURE 2:**
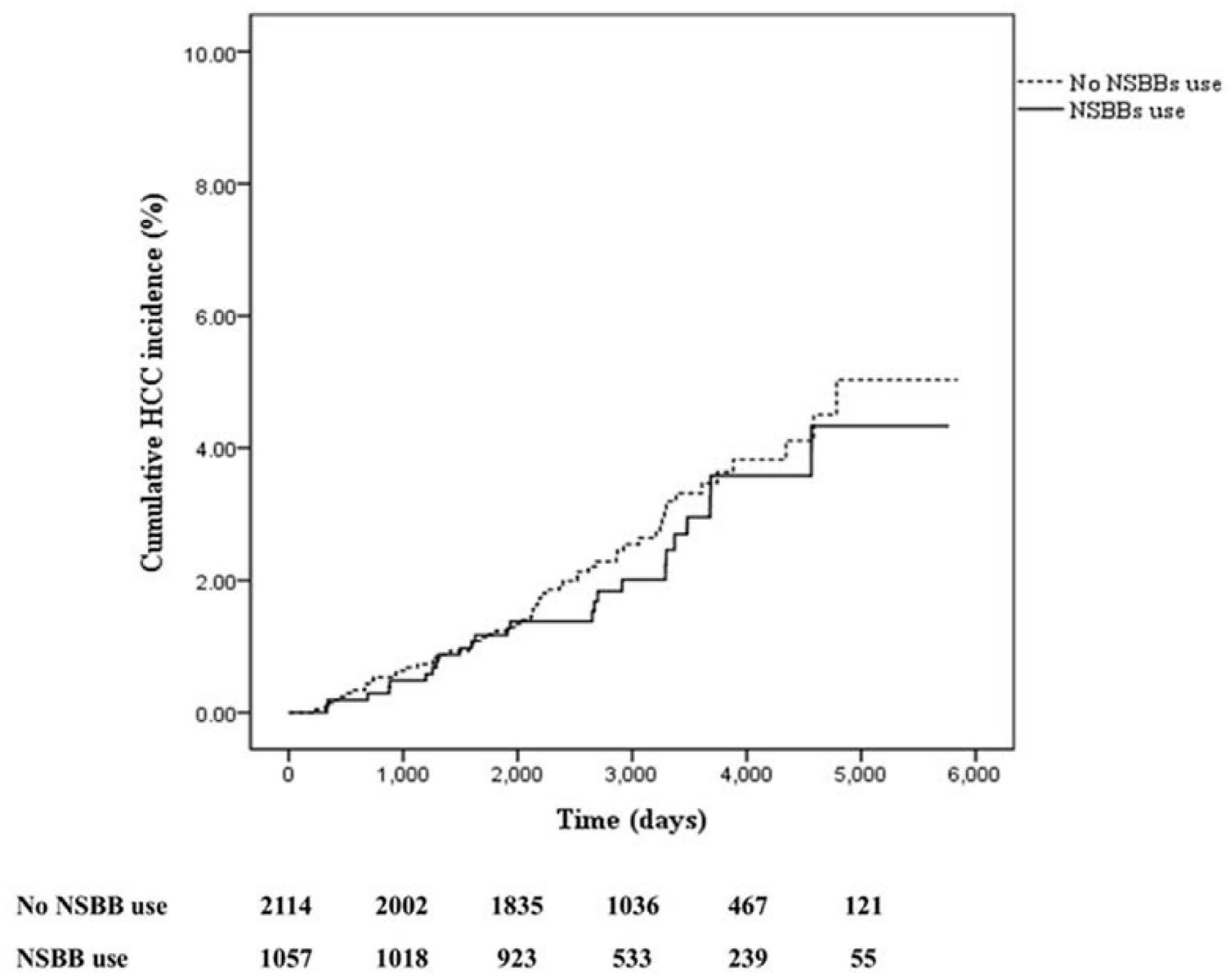
Cumulative incidence of hepatocellular carcinoma. HCC, hepatocellular carcinoma; NSBB, nonselective beta blocker.

Figure 2 presents the cumulative incidence of HCC. No significant difference was observed in the Kaplan–Meier curves between the two groups (*p* = 0.49). The 5-years cumulative incidence of HCC was 1.36% (95% CI, 0.81–2.29) and 1.37% (95% CI, 0.95–1.99) among the NSBB users and nonusers, respectively. The 10-years cumulative incidence of HCC was 3.55% (95% CI, 2.28–5.51) and 3.61% (95% CI, 2.71–4.79) among the NSBB users and nonusers, respectively.

Potential risk factors for HCC were identified on the basis of the results of univariable analysis (Table 2). Old age (HR, 1.04; 95% CI, 1.02–1.05; *p* < 0.01), male sex (HR, 2.00; 95% CI, 1.20–3.34; *p* < 0.01), hypertension (HR, 1.88; 95% CI, 1.18–3.00; *p* < 0.01), diabetes (HR, 2.52; 95% CI, 1.56–4.06; *p* < 0.01), and metformin use (HR, 3.00; 95% CI, 1.80–5.00; *p* < 0.01) were associated with a high risk of HCC. After adjustment for covariates, old age and male sex remained independent risk factors.

**TABLE 2:** Univariable and multivariable Cox regression model analysis for HCC risk.

Variables	Crude HR (95% CI)	*p* value	Adjusted^a^ HR (95% CI)	*p* value
NSBB use vs no NSBB use	0.85 (0.54–1.33)	0.47	0.82 (0.52–1.31)	0.41
Age	1.04 (1.02–1.05)^b^	<0.01	1.03 (1.02–1.05)^b^	<0.01
Sex/male	2.00 (1.20–3.34)^b^	<0.01	2.01 (1.22–3.30)^b^	0.01
Hypertension	1.88 (1.18–3.00)^b^	<0.01	1.21 (0.71–2.09)	0.48
Hyperlipidaemia	0.78 (0.42–1.44)	0.42	0.58 (0.28–1.18)	0.13
Diabetes	2.52 (1.56–4.06)^b^	<0.01	1.44 (0.65–3.19)	0.37
Tobacco use	1.38 (0.19–10.20)	0.75	1.55 (0.19–12.42)	0.68
Alcohol use	—	—	—	—
Obesity	—	—	—	—
Nonalcoholic liver diseases	1.37 (0.34–5.46)	0.65	1.38 (0.34–5.66)	0.66
Antiviral therapy	2.36 (0.60–9.26)	0.21	2.87 (0.72–11.52)	0.14
Statin	1.06 (0.51–2.20)	0.87	0.94 (0.41–2.16)	0.89
Metformin	3.00 (1.80–5.00)^b^	<0.01	2.00 (0.87–4.61)	0.10
Aspirin	1.56 (0.92–2.66)	0.10	1.03 (0.57–1.85)	0.92

HR, hazard ratio; CI, confidence interval; NSBB, nonselective beta blocker; HCC, hepatocellular carcinoma.

a Adjusted for age, sex, hypertension, hyperlipidaemia, diabetes, nonalcoholic liver diseases, antiviral therapy, statin use, metformin use, aspirin use, tobacco use, alcohol use, and obesity (Model 4).

b *p* < 0.05.

We assessed the association between NSBB use and incident HCC by performing multivariable analysis. No significant reduction in HCC risk was observed in the NSBB users compared with the NSBB nonusers, although the risk of HCC appeared to decrease (model 4, HR, 0.85; 95% CI, 0.52–1.38; *p* = 0.50; Table 2). We calculated HRs in different models as follows: Multivariable HRs were 0.80 (95% CI, 0.49–1.28; *p* = 0.35) in model 1, 0.80 (95% CI, 0.50–1.29; *p* = 0.36) in model 2, 0.84 (95% CI, 0.52–1.36; *p* = 0.48) in model 3, and 0.79 (95% CI, 0.50–1.27; *p* = 0.34) in model 5. Additional details regarding statistical analysis are provided in the Supplementary Appendix.

### 3.3 Secondary Outcome

The dose- and duration-dependent associations were not observed between NSBB use and HCC risk (Table 3). The 90–180 cDDD group had a lower adjusted HR (HR, 0.54; 95% CI, 0.17–1.77) than did the <90 cDDD group (HR, 1.08; 95% CI, 0.56–2.05) and ≥180 cDDD group (HR, 0.76; 95% CI, 0.40–1.42). No change in HCC risk was observed among the three groups. The median duration of NSBB use was 1.46 years (interquartile range, 0.61–4.55 years). In terms of duration, HCC risk in the NSBB users was similar to that in the NSBB nonusers (HR, 1.04; 95% CI, 0.54–1.99) among the short-term users (<1 year). Although the adjusted HR considerably decreased in the patients who used NSBBs for ≥5 years, no significant difference was observed between the users and nonusers (HR, 0.50; 95% CI, 0.19–1.37).

**TABLE 3:** Dose and duration of NSBB use and risk of HCC.

Events and subgroup	*n*	HCC (%)	Crude HR (95% CI)	Adjusted HR^a^ (95% CI)
Dose				
No NSBB use	2,114	58 (2.74)	1 [Reference]	1 [Reference]
<90 cDDDs	446	11 (2.47)	0.93 (0.49–1.77)	1.08 (0.56–2.05)
90–180 cDDDs	189	3 (1.59)	0.56 (0.17–1.78)	0.54 (0.17–1.77)
≥180 cDDDs	422	11 (2.61)	0.89 (0.48–1.66)	0.76 (0.40–1.42)
Duration				
No NSBB use	2,114	58 (2.74)	1 [Reference]	1 [Reference]
<1 year	432	11 (2.55)	0.96 (0.51–1.83)	1.04 (0.54–1.99)
1–5 years	392	10 (2.55)	0.92 (0.47–1.78)	0.84 (0.43–1.63)
≥5 years	233	4 (1.72)	0.56 (0.21–1.48)	0.50 (0.19–1.37)

DDDs, defined daily doses; HR, hazard ratio; CI, confidence interval; NSBB, nonselective beta blocker; HCC, hepatocellular carcinoma. *p < 0.05.

a Adjusted for age, sex, hypertension, hyperlipidaemia, diabetes, nonalcoholic liver diseases, antiviral therapy, statin, metformin, aspirin, tobacco use, alcohol use, and obesity.

### 3.4 Pharmacological Classes and Effect of Individual NSBBs

Properties such as pharmacological classes or effects of individual NSBBs did not affect HCC prevention (Table 4). The patients prescribed beta-blocking agents alone exhibited similar HCC risk to that of the nonusers (HR, 0.90; 95% CI, 0.49–1.67). Among those prescribed alpha- and beta-blocking agents, HCC risk did not significantly decrease, although their adjusted HR was lower than that of their matched counterparts (HR, 0.53; 95% CI, 0.21–1.36). In another subgroup analysis, we investigated the effect of major drugs, namely propranolol (*n* = 640, 60.6%) and carvedilol (*n* = 346, 32.7%). The risk of HCC incidence was similar between the propranolol users and their matched nonusers (HR, 0.88; 95% CI, 0.47–1.67). Similarly, the carvedilol users did not have a significantly lower incidence of HCC than did the nonusers (HR, 0.53; 95% CI, 0.19–1.53).

**TABLE 4:** Pharmacological classes of NSBBs and risk of HCC.

Events and subgroup	Events No./Total No. (%)		Crude HR (95% CI)	Adjusted HR (95% CI)
	NSBB use	No NSBB use		
Pharmacologic class				
Only beta-blocking agents	13/592 (2.20)	32/1,184 (2.70)	0.79 (0.42–1.48)	0.90 (0.49–1.67)^a^
Alpha- and beta-blocking agents	6/325 (1.85)	19/650 (2.92)	0.63 (0.26–1.51)	0.53 (0.21–1.36)^b^
Specific drug				
Propranolol	12/568 (2.11)	30/1,136 (2.64)	0.78 (0.41–1.49)	0.88 (0.47–1.67)^c^
Carvedilol	6/266 (2.26)	16/532 (3.01)	0.74 (0.30–1.79)	0.53 (0.19–1.53)^d^

HR, hazard ratio; CI, confidence interval; NSBB, nonselective beta blocker; HCC, hepatocellular carcinoma. *p < 0.05.

a Adjusted for variables SMD > 0.10: sex, hypertension, mental diseases, and tobacco use.

b Adjusted for variables SMD > 0.10: sex, hyperlipidaemia, mental diseases, cardiac diseases, and aspirin use.

c Adjusted for variables SMD > 0.10: sex, hypertension, mental diseases, and tobacco use.

d Adjusted for variables SMD > 0.10: sex, hyperlipidaemia, nonalcoholic liver diseases, mental diseases, cardiac diseases, metformin use, and aspirin use.

### 3.5 Multivariable Stratified Analysis for Aspirin Therapy

In the multivariable stratified analysis, we stratified patients by several baseline characteristics that affect HCC development (Table 5). Most of the values were consistently <1.0 and did not reach statistical significance. The NSBB users had a 51% lower risk of HCC than did the nonusers among the patients aged >55 years (HR, 0.49; 95% CI, 0.25–0.96; *p* = 0.04). However, by contrast with the other subgroups, the NSBB users without hypertension had a slightly higher but not significant risk of HCC than that of the nonusers without hypertension (HR, 1.05; 95% CI, 0.50–2.20).

**TABLE 5:** Multivariate stratified analyses of the association between NSBB use and risk of HCC.

Variable	Events No./Total No		Adjusted HR^a^ (95% CI)	*p* value	Variables for adjustment
	NSBB use	No NSBB use			
Overall	25/1,057	58/2,114	0.82 (0.52–1.30)	0.41	Aspirin use
Age group (years)					
<55	15/621	22/1,284	1.40 (0.74–2.65)	0.30	Tobacco use
≥55	10/436	36/830	0.49 (0.25–0.96)^b^	0.04	Cardiac diseases, asthma, and aspirin use
Sex					
Female	5/437	17/856	0.61 (0.24–1.56)	0.30	Hypertension and metformin use
Male	20/620	41/1,258	0.92 (0.54–1.57)	0.77	Cardiac diseases, statin use, and aspirin use
Hypertension					
No	12/550	19/1,034	1.05 (0.50–2.20)	0.89	Cardiac diseases, tobacco use, and aspirin use
Yes	13/507	39/1,080	0.69 (0.38–1.26)	0.23	Asthma and aspirin use
Hyperlipidaemia					
No	22/855	49/1735	0.87 (0.53–1.41)	0.56	Aspirin use
Yes	3/202	9/379	0.60 (0.16–2.24)	0.45	Cardiac diseases and aspirin use
Diabetes					
No	15/869	42/1757	0.69 (0.39–1.22)	0.20	Aspirin use
Yes	10/188	16/357	0.96 (0.42–2.15)	0.91	Sex, asthma, cardiac diseases, alcohol use, antiviral therapy, and aspirin use
Statin use					
No	24/939	51/1915	0.92 (0.58–1.47)	0.73	Aspirin use
Yes	<3/118	>3/199	0.18 (0.03–1.01)	0.05	Sex, hypertension, hyperlipidaemia, cardiac diseases, asthma, and COPD
Metformin use					
No	18/943	46/1897	0.75 (0.45–1.28)	0.30	Aspirin use
Yes	7/114	12/217	0.92 (0.33–2.52)	0.87	Sex, mental diseases, cardiac diseases, obesity, and aspirin use
Aspirin use					
No	19/857	47/1829	0.84 (0.50–1.41)	0.50	Tobacco use
Yes	6/200	11/285	0.66 (0.24–1.80)	0.41	Sex, hypertension, cardiac diseases, and COPD

HR, hazard ratio; CI, confidence interval; NSBB, nonselective beta blocker; HCC, hepatocellular carcinoma; COPD, chronic obstructive pulmonary disease.

a Adjusted for variables for which SMD, was >0.1 (listed in the column “Variables for adjustment”).

b *p* < 0.05.

### 3.6 Sensitivity Analysis

In our main analysis, although we began the follow-up from the 180th day from the index date to eliminate partial immortal time bias, the time between CHB diagnosis and the first NSBB use was suspected to be the immortal period. The results of sensitivity analysis revealed consistency in the findings even when we reduced immortal time bias by assigning matched dates to the NSBB nonusers on the basis of year of CHB diagnosis (Supplementary Table S1, HR, 0.85; 95% CI, 0.52–1.38). The patients who used selective beta-blockers were excluded because they might have been a potential confounder. The results are in accordance with our earlier observations indicating that HCC risk was not significantly reduced in the NSBB users (HR, 0.67; 95% CI, 0.37–1.20). Lastly, we repeated our analysis with 10 years of follow-up on the basis of the hypothesis that the effects of NSBBs would not last for >10 years. This analysis demonstrated that NSBB use did not reduce the risk of HCC even within 10 years (HR, 0.81; 95% CI, 0.50–1.32).

## 4 Discussion

This nationwide cohort study determined whether the protective effect of NSBBs can affect the risk of HCC in patients with CHB but without advanced liver diseases. Our cohort consisted of patients with CHB but without progress to cirrhosis and decompensation, with the crude HCC incidence rate being 0.31 per 100 person-years; this finding is consistent with that of a study of patients with CHB but not underlying cirrhosis (Do et al., 2014). The results of this study did not reveal an association between NSBB use and a significantly decreased HCC incidence in the patients with CHB.

Studies have indicated a significant association between NSBB use and a significantly lower risk of HCC in patients with cirrhosis (Nkontchou et al., 2012; Herrera et al., 2016; Wijarnpreecha et al., 2021). However, patients with cirrhosis who cannot use NSBBs may have more advanced liver diseases and thus a higher risk of HCC. Therefore, the higher HCC incidence observed in patients with cirrhosis in the non-NSBB group may be associated with severe advanced liver conditions (Huang et al., 2021). This confounding by different disease severity was lower in our study because we included only patients newly diagnosed as having CHB and excluded those with cirrhosis and liver compensation. Patients with cirrhosis had a higher risk of HCC than did those without cirrhosis (Thiele et al., 2014). The haemodynamic-related benefits of NSBBs, such as the reduction in portal hypertension, strongly affect patients with cirrhosis. Therefore, nonhaemodynamic effects, such as HCC prevention, are more prevalent in those with cirrhosis than in those with only CHB (Kim et al., 2012; Hagberg et al., 2016). The results of this study indicated that haemodynamic-related effects might mainly contribute to the protective effects of NSBBs against hepatocarcinogenesis.

Regarding the effect of the dose and duration on the enhancement of causality, only one study analysed the doses of NSBBs and indicated that the protective effect of NSBBs on HCC prevention was observed only in patients receiving a dose of >90 cDDD and not in low-cDDD groups (Yeh et al., 2019). Our findings revealed that the effects of NSBB use on HCC prevention cannot be extrapolated to patients with CHB even under high cumulative doses and increased durations. The results of the subgroup analysis revealed that the carvedilol monotherapy group did not have a significantly lower incidence of HCC than did the nonusers, although the adjusted HR for the carvedilol group was lower than that for the propranolol group. A study demonstrated a significantly lower risk of HCC in patients with cirrhosis by using carvedilol, propranolol, and nadolol (Wijarnpreecha et al., 2021). This finding differs from that of this study possibly because of differences in disease severity between the two studies.

The aetiology in our study population was HBV infection. The process of HCC manifestation in patients with HBV infection differs from that in patients with HCV infection, who mostly develop cirrhosis before HCC (Villanueva, 2019). Studies have reported a more significant protective effect on HCC incidence in patients with HCV- or alcohol-related cirrhosis than in those with HBV-related cirrhosis (Kim et al., 2012; Nkontchou et al., 2012; Herrera et al., 2016; Yeh et al., 2019). The results of our study are consistent with those of a stratified analysis of patients with HBV-associated cirrhosis (Wijarnpreecha et al., 2021). Tolerant doses to NSBBs in different ethnic groups may have resulted in no remarkable effect on HCC prevention in this study. Because the Asian population requires lower doses of NSBBs to reach the target blood pressure and heart rate than does the Caucasian population, the dose used on each day in this study was lower than that used in studies of the Caucasian population (Zhou et al., 1989; Hu et al., 2007). NSBB use appeared to benefit HCC prevention in the Caucasian population more than in the Asian population (Kim et al., 2012; Nkontchou et al., 2012; Herrera et al., 2016; Yeh et al., 2019; Wijarnpreecha et al., 2021).

The results of the multivariable stratified analysis indicated that HCC risk did not significantly decrease regardless of sex, underlying comorbidities, and baseline medication use. However, the patients aged ≥55 years and those using NSBBs had a substantially (51%) lower risk of HCC than did those who did not use NSBBs. By contrast, the patients aged <55 years exhibited no significant difference in HCC risk. This result is in accordance with that of a study conducted in the United States that reported that NSBB use exerted more significant protective effects for HCC in older patients (Wijarnpreecha et al., 2021) Aging is a crucial risk factor for HCC (Yip et al., 2017; Mittal et al., 2018). Thus, this result might be related to mechanisms underlying the effects of NSBB on HCC prevention, including the inhibition of angiogenesis and the beta-adrenergic signaling process, thus resulting in inflammation regulation. The inhibition of angiogenesis and inflammation exerted stronger effects on older patients whose immune system and angiogenic growth were less active than those in younger patients (Moriya and Minamino, 2017). Therefore, a significant reduction in HCC risk might be observed in older patients.

One study reported that nonselective beta blockers may inhibit hepatocarcinogenesis by inhibiting proliferation and inducing apoptosis and S-phase arrest in human liver cancer cell lines (Wang et al., 2018). *In vivo* and *in vitro* studies have demonstrated that beta-adrenergic signaling is associated with inflammation, angiogenesis, apoptosis, cellular replication, DNA damage repair, and cellular immune responses (Ming et al., 1995; Pantziarka et al., 2016; Wang et al., 2018). However, its protective effect was not observed in this study. The differences between this real-world study and experimental studies can be explained by the fact that the doses of NSBBs used in an animal or a cell line in these biological studies are not compatible with those used in humans. The optimal *in vitro* dose of propranolol for anticarcinogenesis in liver cells was 80 μmol/L (80 nmol/ml), which is considerably higher than the 5.3–300 ng/ml (0.02–1.16 nmol/ml) observed in human plasma (Wong et al., 1979; Wang et al., 2018). Therefore, the antitumor effect of NSBBs on liver cancer remains unclear in humans. Numerous pathogenic mechanisms are involved in HBV-associated HCC (Arzumanyan et al., 2013). Hepatocarcinogenesis is not only related to angiogenesis and inflammation, which are the main pathways of anti-HCC effects exerted by NSBBs. Although these processes are inhibited by NSBBs, other mechanisms may lead to the growth of cancer cells, such as the alteration of host gene expression (Nagaya et al., 1987; Tokino et al., 1991).

To the best of our knowledge, this is the first study to comprehensively investigate the effect of NSBBs on HCC prevention in patients with CHB but without cirrhosis and liver decompensation. The confounding by indication bias for advanced liver diseases was reduced in our study. This strong confounder was not allocated appropriately in studies on patients with cirrhosis who had a higher probability of receiving NSBBs for the prevention of oesophageal varices. In addition to avoiding cirrhosis as a confounding factor in our study, we used propensity score matching to ensure the comparability of the indications of NSBBs between the two cohorts. Another strength of this study is that the follow-up period was longer than that of other studies. Because hepatocarcinogenesis gradually advances, a short-term follow-up might not be sufficient for clinical applications. In addition, the LGTD 2000 used as the data source in this study consisted of updated records regarding prescriptions and diagnoses. Thus, we adjusted covariates for medication use, which had been ignored in some studies. Furthermore, our analyses included potentially chemopreventive drugs such as statins, aspirin, metformin, NA, and interferons. Lastly, we used an exposure window to minimise misclassification and ensure new user design. Several sensitivity analyses were performed to eliminate the epidemiological bias related to immortal time, potential confounding by selective beta-blockers, and unclear duration of the effect of NSBBs on cancer prevention.

This study has several limitations that should be addressed. First, this study was limited by the lack of information regarding laboratory data such as serological markers (e.g., HBV DNA levels, HBeAg, and HBsAg) and the genotype of HBV. However, we adjusted for antiviral therapy, which can serve as a surrogate variable for viral load. In addition, we adjusted for alcohol use, tobacco use, and obesity on the basis of related diagnoses; however, patients who were not hospitalised or did not consult a doctor for these problems were not identified. Nevertheless, patients with these diagnoses were considered to have more severe diseases than did those without related diagnoses. Regarding lifestyle factors, information regarding coffee consumption and aflatoxin exposure was lacking. Other information such as patients’ background and behaviour was not recorded, for example, family history and actual adherence to medication and screening. Lastly, the generalizability of these results is subject to certain limitations because most controls were not included in our studies after propensity score matching. However, the similarity of HCC incidence between the overall population in our studies and that in other studies partly supports generalizability.

In conclusion, this study revealed that NSBB use was not associated with decreased HCC occurrence in a nationwide population of patients with CHB. Our findings suggested that the effect of NSBBs on HCC chemoprevention cannot be extrapolated to patients with CHB, although the benefits were observed in patients with cirrhosis in some studies. The remarkable protective effect was only noted in the subgroup of patients aged >55 years. This study provided one of valuable results that it is not clinically required to use NSBBs as recommended chemoprevention for HCC in high-risk patients who have CHB. To evaluate the benefits of NSBBs for HCC prevention, additional studies should be conducted to investigate the anti-HCC effects of NSBBs on high-risk patients.

## Data Availability

The data analyzed in this study is subject to the following licenses/restrictions: The data underlying this study are from National Health Insurance Research Database (NHIRD) and can be accessed by request to the Health and Welfare Data Science Center (HWDC) Health and Welfare, Taiwan (http://dep.mohw.gov.tw/DOS/np-2497-113.html) using the information outlined in the *Methods* section. All applications are reviewed for approval of data release and applicants must follow the Computer-Processed Personal Data Protection Law (http://www.winklerpartners.com/?p=987) and related regulations of National Health Insurance Administration. Requests to access these datasets should be directed to http://dep.mohw.gov.tw/DOS/np-2497-113.html.
