# Supplementary material for: Association Between Nonselective Beta-Blocker Use and Hepatocellular Carcinoma in Patients With Chronic Hepatitis B Without Cirrhosis and Decompensation
Source: Front Pharmacol. 2022 Jan 7;12:805318. doi: 10.3389/fphar.2021.805318 (PMC8777254; doi:10.3389/fphar.2021.805318)

Supplementary Material

**Supplementary Table S1.** Sensitivity analysis with matched index dates in the NSBB nonuser cohort: multivariable Cox regression model analysis

| **Model** | **NSBB treated** | **NSBB untreated** | ***P* value** |
| --- | --- | --- | --- |
| Unadjusted, crude HR (95% CI) | 0.80 (0.50–1.28) | 1 [Reference] | 0.35 |
| Model 1, adjusted HR (95% CI) ^a^ | 0.80 (0.49–1.28) | 1 [Reference] | 0.35 |
| Model 2, adjusted HR (95% CI) ^b^ | 0.80 (0.50–1.29) | 1 [Reference] | 0.36 |
| Model 3, adjusted HR (95% CI) ^c^ | 0.84 (0.52–1.36) | 1 [Reference] | 0.48 |
| Model 4, adjusted HR (95% CI) ^d^ | 0.85 (0.52–1.38) | 1 [Reference] | 0.50 |
| Model 5, adjusted HR (95% CI) ^e^ | 0.79 (0.50–1.27) | 1 [Reference] | 0.34 |
| HR, hazard ratio; CI, confidence interval; NSBB, nonselective beta-blocker; HCC, hepatocellular carcinoma.  **P* < 0.05 | | | |
| ^a^Model 1 adjusted for continuous years from CHB diagnosis, age, and sex. | | | |
| ^b^Model 2 adjusted for continuous years from CHB diagnosis, age, sex, hypertension,  ^b^ hyperlipidaemia, diabetes, and nonalcoholic liver diseases. | | | |
| ^c^Model 3 adjusted for continuous years from CHB diagnosis, age, sex, hypertension,  ^c^ hyperlipidaemia, diabetes, antiviral therapy, statin use, metformin use, and aspirin use. | | | |
| ^d^Model 4 adjusted for continuous years from CHB diagnosis, age, sex, hypertension, hyperlipidaemia, diabetes, nonalcoholic liver diseases, antiviral therapy, statin use, metformin use, aspirin use, tobacco use, alcohol use, and obesity. | | | |
| ^e^Model 5 adjusted for continuous years from CHB diagnosis and aspirin use. | | | |

**Supplementary Figure S1.** Study design


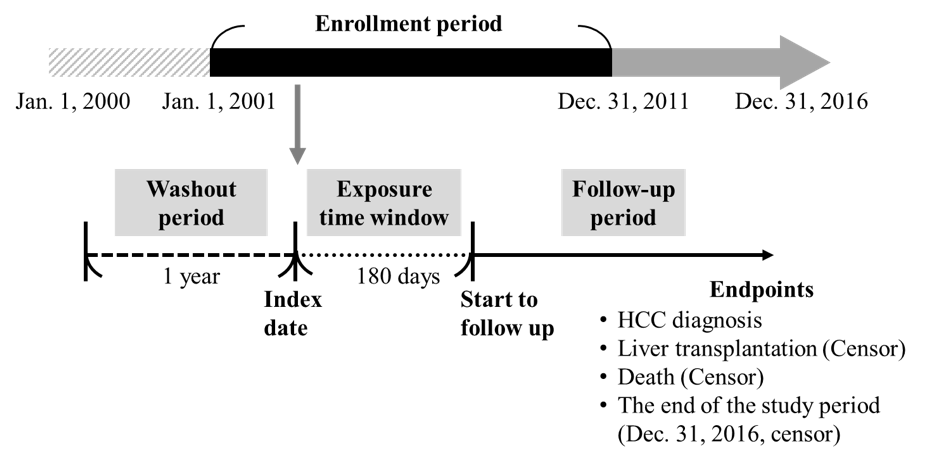

Supplement: Supplementary file 1 [file DataSheet1.docx]
